# How do inside directors affect corporate R&D investment? The moderating role of CEO equity incentives

**DOI:** 10.1371/journal.pone.0317123

**Published:** 2025-02-07

**Authors:** Jianqing Zhou, Yulian Peng

**Affiliations:** 1 School of Finance and Economics, Guangdong Polytechnic Normal University, Guangzhou, China; 2 College of Management, Guangzhou City University of Technology, Guangzhou , China; 3 Guangdong Education Big Data Research Center, Guangdong University of Technology, Guangzhou , China; University of Bologna, ITALY

## Abstract

This study aims to reveal whether non-CEO inside directors can promote corporate research and development (R&D) investment. Using a panel data of 3,002 Chinese manufacturing listed firms from 2011 to 2021, we find that inside directors can significantly promote corporate R&D investment. We also find that when the CEO has equity incentives to alleviate agency conflicts, the role of inside directors in promoting R&D investment is significantly weakened. Additional analysis show that the promotive effect of inside directors on R&D investment is significant only in samples of non-state-owned enterprises (non-SOEs), male CEOs, older CEOs, and small boards, but not for the samples of state-owned enterprises (SOEs), female CEOs, younger CEOs, and large boards. These findings extend the scope of research on the economic consequences of inside directors and have important implications for the optimization and adjustment of corporate governance policies.

## 1. Introduction

Corporate R&D investment is the prerequisite for technological innovation and an important source of increasing market share, improving product value, and obtaining sustainable competitive advantages [1]. Principal-agent theory believes that the most important agency issue is not that CEO occupy corporate resources in the form of self-benefits, but they underinvest in technological innovation due to risk aversion, which will damage corporate viability and lead to a continuous decline in corporate value [2]. In the past decade, the exploration of the driving factors for firms to invest in R&D has always been an important topic in corporate governance research. Studies have generally shown that the boards of directors can monitor the CEO’s opportunistic behavior and play an important role in influencing the corporate R&D investment [3]. However, previous studies only emphasized the influence of outside or independent directors on corporate R&D investment [4,5], while ignored the potential role of non-CEO inside directors (who concurrently serve as members of the firm’s board of directors and management team). Therefore, our paper aims to fill this gap to some extent by investigating whether and how inside directors influence corporate R&D investment.

Traditional governance theory often emphasizes the supervisory role of board independence in mitigating the CEO’s tendencies toward risk aversion and insufficient innovation, while exhibiting a stance of distrust and vigilance towards inside directors [6,7]. Due to the fact that inside directors are members of the executive team led by the CEO, their career advancement and compensation levels are largely contingent upon the evaluation and recognition by the CEO. Consequently, inside directors are likely to develop a collusive relationship with the CEO, which complicates their ability to effectively monitor the CEO’s opportunistic behaviors [8,9]. For this reason, a substantial number of scholars have explored the relationship between outside or independent directors and corporate R&D investment. However, no consensus has been reached. Some studies have indicated that outside or independent directors play a significant role in supvising the risk aversion tendencies of CEOs, thereby contributing positively to corporate risk-taking, strategic transformation, technological innovation, and R&D investments [10–12]. Conversely, other research has found that outside or independent directors, due to insufficient information, lack the capacity to effectively supervise CEOs, thereby exerting a significant inhibitory effect on corporate R&D investments [4].

In fact, as early as the beginning of this century, scholars expressed skepticism regarding the governance effects of board independence, introducing the concept of the “independence paradox” [13]. They argued that outside or independent directors may need to rely on information provided by managers for decision-making, which could potentially render them susceptible to manipulation by managers [13]. On the contrary, inside directors, due to their direct involvement in the operational activities of the firm, possess a deeper understanding of specific internal information and should be given due consideration. By alleviating information asymmetry, they contribute to the oversight of the CEO and enhance the governance quality of the board of directors [14]. In recent years, numerous studies have indicated that inside directors play a positive supervisory governance role in areas such as CEO succession [15], corporate social responsibility management [16], and the efficiency of internal controls [17]. Therefore, the question arises: can inside directors exert a similar positive impact in the realm of corporate R&D investment? This is an issue that warrants further in-depth investigation.

At the same time, corporagte governance mechanisms for agency conflicts often include two types: supervision and incentives. It is worth noting that corporate governance mechanisms do not exist in isolation, in the process of analyzing corporate behavior, the impact of board supervision and CEO incentives should be considered comprehensively [18]. When scholars discuss how to deal with the agency issue of corporate R&D investment, in addition to the supervisory effect of the board, they also emphasize the incentive effect for the CEO [19]. A large number of empirical studies have shown that CEO equity incentives can alleviate agency conflicts and promote R&D investment [20,21]. The reason is that CEO shareholding makes the CEO both a manager and an owner of the firms, which helps narrow the interest gap between the principal and the agent. Therefore, when we discuss the impact of inside directors on R&D investment, we cannot ignore the joint impact of CEO equity incentives.

The boards of directors of Chinese firms generally consist of independent directors, non-executive directors and inside directors, with the former two categorized as outside directors. Among these, non-executive directors are typically appointed by major shareholders as representatives of their equity interests. They do not engage in business operations but safeguard the interests of major shareholders through the decision-making progress of the board of directors. To ensure the independence of the board of directors, the Chinese government mandates that the number of inside directors within listed firms should not exceed 50% of the total number of board members. Specifically, the China Securities Regulatory Commission issued the “Guiding Opinions on Establishing an Independent Director System in Listed Firms” and the “Guidelines on the Articles of Association of Listed Firms” in 2001 and 2006 respectively, requiring that independent directors shall not be less than 1/3 and that inside directors shall not be more than 1/2. The practice of restricting inside directors is fundamentally similar to that in many countries, exhibiting a certain degree of representativeness and typicality [22]. Meanwhile, the manufacturing sector exhibits a strong demand for technological innovation, serving as the primary initiator and beneficiary of R&D investments. Since 2010, China has emerged as the world’s largest manufacturing nation, providing a substantial number of research sample for the study of R&D investments [21].

Therefore, we use a large sample dataset of 3002 Chinese manufacturing listed firms from 2011 to 2021 to empirically examine the impact of inside directors on corporate R&D investment, as well as the moderating effect of CEO equity incentives. Our research findings indicate that inside directors can effectively supervising the opportunistic behaviors of the CEO, thereby exerting a positive influence on corporate R&D investments. Concurrently, the equity incentives for the CEO exhibits a substitutive relationship with inside directors in terms of innovative governance effect, thereby significantly diminishing the facilitative role of inside directors in promoting R&D investments. Furthermore, due to the influence of inside directors on the behavior of the CEO is related to their corporate environment, the moral hazard of the CEO, and the governance efficiency of the board of directors, we also find that the promotive effect of inside directors on R&D investment exhibits significant variability based on the nature of the firm, the gender and age of the CEO, as well as the size of the board of directors.

The contributions of this study are primarily reflected in the following aspects. First, it expands the research on the driving factors of corporate R&D investment from the new perspective of inside directors. Previous research, grounded in considerations of board independence, has often focused on the influence of outside or independent directors on corporate R&D investment [4,23]. In contrast, our study emphasizes the significant role of inside directors in the process of corporate R&D investment. The results of this study validate the positive governance effect of inside directors, contributing to the reversal of the negative perception that inside directors are entirely untrustworthy, and thereby providing theoretical references for optimizing the structure of corporate boards [13].

Secondly, our paper reveals the bundling effect of innovative governance mechanisms. To prevent insufficient innovation by managers, agency theory posits that both board supervision and CEO incentives serve as governance mechanisms that can positively influence corporate innovation [24]. However, existing research predominantly focuses on the innovative impact effects of board supervision or CEO incentives in isolation, without integrating the two aspects for comprehensive consideration. [4,25]. Our paper combines the supervision of inside directors with the incentives of CEO, and finds that they exhibit a substitutive effect rather than a complementary effect in promoting R&D investment. This research finding carries significant implications for preventing “over-governance” [26].

Finally, our paper delineates the boundary conditions of the innovative governance effects of inside directors. In the heterogeneity analysis, we further reveals that the positive influence of inside directors on R&D investment is significant only within the samples of non-SOEs, male CEOs, older CEOs, and smaller boards, while it is not significant in the samples of SOEs, female CEOs, younger CEOs, and larger boards. This research finding not only enriches the existing research framework but also holds significant practical implications for the tailored customization and flexible adjustment of corporate governance policies.

The subsequent parts of this paper are organized as follows. Section 2 outlines the relevant literature and develops research hypotheses. Section 3 describes the research design. Section 4 reports the empirical results. Section 5 presents the heterogeneity analysis. Section 6 discusses the research findings, and Section 7 concludes the paper.

## 2. Literature review and research hypotheses

### 2.1. CEO’s insufficient innovation and the governance role of the board of directors

The essence of technological innovation is a long process of trial and error. The contingency of successful innovation brings huge risks to R&D investment projects, and large investments in R&D cannot guarantee safe and stable income and returns in the future [27]. As the leader of the firm’s top management team, the CEO has a vital influence on the firm’s long-term development strategy and plays a decisive role in the decision-making process of corporate R&D investment [28]. Unlike shareholders who pursue excess profits and the long-term development of the firm, CEOs tend to be more risk-averse and resistant to technological innovation for the following reasons. Firstly, the CEO is required to undergo annual performance evaluations, and an increase in R&D investment may lead to a decrease in the firm’s profits. To prevent the erosion of CEO compensation and benefits, they may opt to reduce R&D investments in order to enhance the firm’s short-term performance [29]. Secondly, the high failure risk associated with innovation may result in the bankruptcy or acquisition of firms. In such context, CEOs may be motivated to reduce long-term R&D investments in order to safeguard their positions [30]. Thirdly, certain stakeholders, such as institutional investors, tend to prioritize short-term earnings over long-term assets in order to profit from fluctuations in stock prices. They may exert pressure on the CEO to reduce R&D investments in order to increase short-term profits [31]. Therefore, in the context of poor corporate governance, once a CEO acquires control power, they are likely to reduce the level of R&D investment [32].

As the supervisory department of the top management team, the board of directors plays an irreplaceable role in preventing the CEO’s insufficient innovation. Previous research has shown that director characteristics can have an important impact on corporate R&D investment, such as director’s gender, director’s education background, as well as director’s human capital and relational capital [33]. More relevant to our paper, some scholars have explored the impact of independent directors and outside directors on corporate R&D investment and have formed rich research conclusions.

Since the emergence of a series of accounting scandals at the beginning of this century, there has been nearly universal support for the notion that corporate boards must maintain sufficient independence to effectively supervise managerial misconduct [7]. Due to the fact that outside or independent directors do not hold positions within the firm and their compensation is primarily not dependent on the firm, they are better positioned to effectively monitor the opportunistic behaviors of management. Numerous studies have demonstrated that outside or independent directors can enhance the supervision of CEO in terms of insufficient innovation, thereby facilitating the advancement of corporate R&D investment activities [6,23]. However, some scholars have proposed the concept of the “independence paradox,” arguing that outside or independent directors are, in fact, unable to effectively supervise managers because the information they require comes from the managers themselves, which may render them susceptible to manipulation by those managers [13]. Therefore, outside directors may be unable to effectively monitor the CEO’s insufficient innovation due to a lack of information, which could have a negative impact on corporate R&D investments [4].

In light of these contradictory research findings, we contend that solely focusing on the independence of the board, while neglecting the significant role of inside directors, does not provide a comprehensive understanding of the governance effects of the board structure. Inside directors possess substantial informational advantages and influence, enabling them to significantly impact corporate strategic decision-making [7]. Therefore, in analyzing the relationship between the board of directors and R&D investment, it is essential to delve into the impact of inside directors.

### 2.2. The impact of inside directors on corporate R&D investment

Traditional agency theory holds that the CEO is the leader of the firm’s top management team, inside directors are members of the top management team, and the career development and salary levels of inside directors largely depend on the CEO’s evaluation and recognition. Therefore, inside directors not only need to work to improve corporate operating efficiency, but may also need to assist the CEO in resisting board supervision to achieve his or her private goals [34]. In other words, the governance effect of the board depends on the power game between directors and managers, and inside directors can enhance the power of the CEO and weaken the supervisory function of the board. In this sense, inside directors are related to poor corporate governance, which is not conducive to preventing the CEO’s opportunistic behavior of insufficient innovation.

However, the reality may not be so straightforward. Inside directors possess the prerequisite, motivation, and capability to supervise the insufficient innovation of the CEO, thereby facilitating the advancement of corporate R&D investment activities.

Firstly, inside directors possess a comprehensive understanding of the firm’s internal information, which serves as a prerequisite for effectively supervise the CEO. Unlike outside directors, who lack sufficient understanding of internal information and thus cannot effectively supervise management, inside directors participate in the daily operations and strategic decision-making, enabling them to acquire privileged information within the firm [35]. Therefore, inside directors are able to mitigate the information asymmetry between the board and management, thereby enhancing the level of corporate governance [14]. In this context, in the face of insufficient innovation from the CEO, inside directors possesse the prerequisites for supervise the CEO.

Secondly, inside directors have the motivation to supervise the CEO. On the one hand, inside directors, as members of the board of directors, are required to fulfill the job responsibilities and societal expectations associated with their role as directors. Therefore, inside directors have the responsibility to supervise the opportunistic behavior of the CEO [36]. On the other hand, out of concern for career prospects, inside directors are more inclined to safeguard the firm’s long-term interests, thereby helping to supervise the CEO’s opportunistic behavior. Relevant research on human resources shows that inside directors are often regarded as the best candidates to succeed the CEO, and the sustainable development of the firm can bring good career development prospects to inside directors [37]. On the contrary, if a firm experiences poor performance or growth difficulties, the professional reputation of inside directors will be damaged accordingly, and they may lose their board seats in their own firm and other firms [38]. Additionally, compared to outside directors, inside directors possess a greater understanding of technological information and innovation processes, which enhances their confidence in technological innovation [39], leading to a lower perception of risk associated with R&D investments. Therefore, in the context of insufficient innovation by the CEO, inside directors are motivated to constrain the opportunistic behaviors of the CEO and promote corporate R&D investments due to their job responsibilities, career prospects, and greater confidence.

Thirdly, inside directors possess the capacity to supervise the CEO. Although managers are generally reluctant to share information about the CEO’s opportunistic behavior with the board, because doing so may be threatened by the CEO and hinder their career development. However, once managers become board members, they will be able to report directly to the board, making it easier to gain board support and be able to deal with potential threats from the CEO. In this case, inside directors are able to share information with other directors, thereby helping to monitor the CEO’s opportunistic behavior [40]. Research shows that when inside managers establish connections with outside directors, it facilitates information communication and improves the governance efficiency of the board [41]. Muslu [42] took large European firms as a sample and empirical research showed that firms with more inside directors have higher levels of corporate governance.

In summary, the theoretical analysis above shows that inside directors are different from other managers. As board members, they possess the prerequisites, motivation, and capability to supervise the CEO’s opportunistic behavior, and are expected to have a positive impact on corporate R&D investment. Therefore, we propose the following research hypotheses:

H1: Inside directors can promote corporate R&D investment, all else being equal.

### 2.3. The moderating effect of CEO equity incentives

Agency issues arise from information asymmetry and goal conflicts between shareholders and management [2]. Therefore, corporate governance research to deal with agency conflicts is often based on two mechanisms: supervision and incentives. Strengthening the level of board supervision to reduce information asymmetry or implementing effective incentives for managers to reduce goal conflicts are common governance mechanisms within firms [24]. It is important to note that the two types of corporate governance mechanisms do not exist in isolation [26]. In the process of studying the impact of governance mechanisms on corporate business decisions, it is necessary to consider the joint impact of supervision mechanisms and incentive mechanisms [18]. Therefore, when examining how inside directors supervise the insufficient innovation of the CEO, the joint effect of CEO incentives cannot be ignored.

From the perspective of CEO incentives, previous research has shown that aim-based short-term CEO compensation has a significant negative impact on R&D investment [43]. On the contrary, CEO equity incentives can help overcome managers’ short-termism and make them pay more attention to the firm’s long-term development, thereby promoting corporate R&D investment [20]. On the one hand, CEO equity incentives can promote the consistency of goals between principals and agents, making the CEO more inclined to protect rather than harm the interests of shareholders during strategic decision-making [44]. On the other hand, since technological innovation is more in line with the long-term interests of shareholders, when CEOs hold corporate stocks, they will be more willing to take risks and actively carry out R&D investment activities [45]. Therefore, the existing literature has largely reached a consensus that, CEO equity incentives can effectively curb opportunistic behaviors of insufficient innovation and promote corporate R&D investments [24].

The corporate governance mechanism is interrelated rather than existing in isolation. The joint governance effects of board supervision and CEO incentives primarily manifests in two types of relationships: complementary and substitution [46]. The complementary effect refers to the mutual reinforcement between supervisory effects and incentive effects, whereby their combination can further enhance the level of corporate governance. Research has indicated that there exists a complementary relationship between board supervision and CEO incentives within the realm of internal control and earnings management [47,48]. The substitution effect refers to the governance role of board supervision and CEO incentives acting as substitutes for one another. When incentives are insufficient, the supervisory effect is likely to be stronger; conversely, when incentives are adequately provided, the supervisory effect tends to diminish. A study by Lim [20] found that the impact of CEO restricted stock incentives on R&D investments is mitigated by the heightened vigilance of the board. Meanwhile, a study by Zona [19] also found that the positive impact of CEO stock options on R&D investment is mitigated by board monitoring.

According to this research stream, if CEOs have equity incentives, it will help alleviate their opportunistic behavior, and will improve the level of corporate R&D investment. In this case, the CEO does not have serious opportunistic behavior in technological innovation, so we expect that the marginal contribution of the supervisory role of inside directors will be significantly reduced. In other words, for corporate R&D investment, the supervisory role of inside directors and the equity incentive role of CEO are substitutes for each other. For this reason, we put forward the second research hypothesis.

H2: CEO equity incentives can weaken the promotive effect of inside directors on corporate R&D investment, all else being equal.

## 3. Research design

### 3.1. Institutional setting and data collection

#### (1) Institutional setting.

Since the implementation of corporate governance policies based on agency theory has been mandated by legislation (e.g., Sarbanes-Oxley Act of 2002), there has been a heightened vigilance regarding inside directors and a pursuit of independence within corporate boards of directors [22]. Compared to inside directors, outside directors are not members of the firm’s top management team and do not engage in daily management, thereby exhibiting a greater degree of independence [49]. In order to ensure the independence of the board of directors, the China Securities Regulatory Commission mandates that the proportion of inside directors in listed firms shall not exceed 50%. Although the practice of limiting the number of inside directors is relatively mainstream and aligns with the traditional agency theory, its effectiveness in promoting corporate R&D investment requires further empirical investigation.

Since 2010, China has emerged as the largest manufacturing country in the world, hosting a substantial number of listed manufacturing firms. Meanwhile, Chinese manufacturing firms are undergoing a period of transformation, striving to achieve a transition from traditional manufacturing to advanced manufacturing through technological innovation. In 2015, the Chinese government issued the “Made in China 2025” initiative, which aims to achieve the upgrading of traditional industries and sustainable economic development through technological innovation. It is evident that China’s manufacturing sector not only comprises a significant number of listed firms but also actively engages in technological innovation activities, thereby providing ample and appropriate samples for research on corporate R&D investment [50].

#### (2) Data collection.

Given that the manufacturing sector is the primary source of corporate technological innovation, we refer to the study by Chang et al [21] and focus on manufacturing firms to explore issues related to corporate R&D investment. At the same time, since 2010, China’s manufacturing output has ranked first in the world. Following the research conducted by Sun et al. [51], we have defined the sample period as spanning from 2011 to 2021. Relevant data on inside directors, CEO equity incentives, corporate R&D investment, and control variables are extracted from the China Stock Market & Accounting Research Database (CSMAR). CSMAR is an economic and financial database developed based on the professional standards of international authoritative databases such as CRSP, Compustat, TAQ, and Thomson, and combined with China’s specific conditions. It is widely used in academic research on China’s economic issues, demonstrating sufficient professionalism and reliability.

During the data collection process, in order to enhance the robustness of this study, we excluded samples of firms designated as Special Treatment (ST and *ST), which indicate anomalous financial conditions or significant delisting risks. Additionally, we also excluded samples with missing data for key variables. Ultimately, we obtained a total of 20,906 firm-year observations from 3,002 listed firms. In order to mitigate the adverse effects of outliers, we conducted winsorization at the 1st and 99th percentiles to the continuous variables.

### 3.2. Variables

#### (1) Dependent variable: corporate R&D investment.

We use R&D intensity to measure corporate R&D investment. Specifically, following the research of Shaikh et al [52], we use R&D expenditures as a percentage of sales to measure R&D investment (R&D_S). This measurement indicates the proportion of annual sales that a firm allocates to R&D projects, reflecting the firm’s commitment to technological innovation. We follow previous research habits and replace missing R&D expenditures with zero, because according to Chinese accounting standards, firms must report R&D expenditures every year [20]. For the dependent variable with missing values retained, we denote it as R&D_M, while for the dependent variable with missing values replaced by zero, we denote it as R&D_S.

#### (2) Independent variable: inside directors.

According to China’s “Company Law”, the board of directors adopts a one-person-one-vote system for decision-making, and each director has an equally important impact on corporate strategy. Therefore, the composition of the board can be used to measure the influence of inside directors. Considering that CEOs are often board members, the measurement of inside directors needs to exclude the CEO to reflect the supervisory role of inside directors. Following the study by Lin et al. [17], we use the proportion of non-CEO inside directors (PID) to measure inside directors. In addition, to enhance the robustness of this study, we also measure inside directors by the number of non-CEO inside directors (NID).

#### (3) Moderating variable: CEO equity incentives.

In order to more fully reflect the degree of common interests between the CEO and shareholders, we considered the cumulative effect of equity incentives. Following the research of Zhao and Lin [53], we measure CEO equity incentives (EI) using the number of CEO’s shareholdings. The larger the CEO’s shareholding, the smaller the agency conflict between the CEO and shareholders, and it is expected that the CEO will be more willing to take innovation risks and invest in R&D.

#### (4) Control variables.

We include a series of control variables from three aspects: firm characteristics, CEO characteristics, and government subsidies. These variables are considered to be influencing factors of corporate R&D investment [52,54]. Firstly, firm characteristics include firm size (Size), financial leverage (Lever), growth capability (Growth), cash slack (Cash), and ownership concentration (OC). Firm size is the natural logarithm of the firm’s total assets. Financial leverage is the firm’s debt-to-asset ratio. Growth capability is the firm’s sales growth rate. Cash slack is the ratio of monetary funds to total assets. Ownership concentration is the percentage of shares held by the largest shareholder. Secondly, CEO characteristics include CEO gender (Gender) and CEO age (Age). The value of Gender is 1 if the CEO is male and 0 if the CEO is female. Age is represented by the CEO’s age. Finally, we also control for government subsidies (Subsidy), expressed as the percentage of government subsidies received by a firm to its total assets. At the same time, we further control the industry dummy variable (Ind) and the year dummy variable (Year). The specific variable description is shown in Table 1.

**Table 1:** Variable definition.

Type	Symbol	Definition
Dependent variable	*R&D_S*	R&D expenditure as a percentage of sales.
Independent variables	*PID*	Proportion of non-CEO inside directors.
	*NID*	Number of non-CEO inside directors.
Moderator variable	*EI*	Number of CEO’s shareholdings (millions)
Control variables	*Size*	The natural logarithm of the firm’s total assets.
	*Lever*	Debt-to-asset ratio of the firm.
	*Growth*	Sales growth rate of the firm.
	*Cash*	The ratio of monetary funds to total assets.
	*OC*	The percentage of shares held by the largest shareholder.
	*Gender*	Equals 1 if the CEO is male, and 0 if the CEO is female.
	*Age*	CEO’s age.
	*Subsidy*	The percentage of government subsidies received by a firm to its total assets
	*Ind*	A collection of industry dummy variables, equals 1 for the specified industry and 0 for the others.
	*Year*	A collection of year dummy variables, equals 1 for the specified year and 0 for the others.

### 3.3. Analytical approach

To test the research hypotheses of this paper, and to explore the relationship between inside directors, CEO equity incentives, and corporate R&D investment, we employ a panel data regression analysis method, referencing the study by Jin and Li [55]. Panel data not only possesses a larger sample size that can enhance the precision of regression analyses, but also aids in mitigating the issues associated with omitted variable bias. At the same time, panel data can provide dynamic information on individual behaviors, facilitating the resolution of reverse causality issues.

Specifically, we construct the following model to empirically test the impact of inside director on corporate R&D investment, as shown in Eq. (1).


$$R\&D_{it}=\beta_{0}+\beta_{1}ID_{it}+\sum \beta_{i}Controls_{it}+\sum Ind+\sum Year+\varepsilon_{it}$$ (1)

The dependent variable $R\&D_{it}$ represents the R&D investment of firm i in year t, measured by the percentage of R&D expenditure to sales$(R\&D\_S_{it}).$The independent variable $ID_{it}$ represents the size of inside directors, measured using the proportion$\left(PID_{it}\right)$and number $\left(NID_{it}\right)$of inside directors respectively. $Controls_{it}$ represents all control variables, and the specific measurement methods are shown in Table 1. ∑Ind and ∑Year represent dummy variables for industry and year respectively. We focus on the regression coefficient of $ID_{it}$. If $\beta_{1}$ is significantly positive, the hypothesis H1 is established, that is, inside directors can promote corporate R&D investment.

At the same time, in order to test the moderating effect of CEO equity incentives, we construct a regression model as shown in Eq. (2).


$$R\&D_{it}=\beta_{0}+\beta_{1}ID_{it}+\beta_{2}EI_{it}+\beta_{3}ID_{it}\times EI_{it}+\sum \beta_{i}Controls_{it}+\sum Ind+\sum Year+\varepsilon_{it}$$ (2)

In this equation, $EI_{it}$ represents CEO equity incentive, $ID_{it}\times EI_{it}$ represents the interaction term between inside directors and CEO equity incentive, and other control variables are consistent with Eq. (1). We focus on the regression coefficient of the interaction term. If $\beta_{3}$ is significantly negative, then hypothesis H2 is established, that is, CEO equity incentives exert a negative moderating effect on the relationship between inside directors and corporate R&D investment.

## 4. Empirical results

### 4.1. Descriptive statistics

Table 2 reports the descriptive statistics of the relevant variables used in this paper. For Chinese manufacturing listed firms, R&D expenditures accounted for an average of 4.46% of sales revenue. It implies that many Chinese firms have recognized the importance of technological innovation and have vigorously carried out R&D investment activities in recent years to implement innovation-driven development strategies. For the independent variable, the average number of non-CEO inside directors in our sample is 1.32, accounting for 15.5% of the board of directors. It shows that in addition to CEOs, only a limited number of managers concurrently hold positions as directors. In the context of advocating for the independence of the board, inside directors have become a minority within the board.

**Table 2:** Descriptive statistics of main variables.

Variables	Mean	Std. Dev.	Min	Med	Max
R&D_S	4.46	4.00	0	3.70	23.9
PID	0.155	0.122	0	0.143	0.6
NID	1.32	1.08	0	1	6
EI	2.33	5.56	0	0.219	34
Size	22	1.160	19.800	21.80	25.50
Level	0.389	0.200	0.0507	0.375	0.929
Growth	0.172	0.367	−0.527	0.117	2.250
Cash	0.165	0.129	0.00934	0.127	0.625
OC	33.7	14.1	8.98	31.6	73.1
Gender	0.935	0.247	0	1	1
Age	49.9	6.88	32	50	67
Subsidy	0.681	0.727	0.000242	0.456	4.210

### 4.2. Hypothesis tests

To test the two research hypotheses presented in this paper, we employed a stepwise regression analysis, with the results presented in Table 3. Specifically, the regression model in column (1) includes only industry and year dummy variables, aside from the independent variable of inside directors (PID). The regression model in column (2) builds upon that in column (1) by incorporating a series of control variables across three dimensions: firm characteristics, CEO characteristics, and government subsidies. The regression model in column (3) builds upon that in column (2) by incorporating CEO equity incentives (EI) and the interaction term between inside directors and CEO equity incentives (PID×EI). To mitigate potential autocorrelation issues within the data, we employed cluster-robust standard errors at the firm level in our regression analysis.

**Table 3:** Hypothesis tests: stepwise regression analysis.

Variables	(1)	(2)	(3)
	R&D_S	R&D_S	R&D_S
PID	0.591**	0.707***	0.870***
	(2.49)	(2.96)	(3.46)
EI			0.020
			(1.63)
PID×EI			−0.078*
			(−1.67)
Size		0.055	0.054
		(0.69)	(0.67)
Level		−1.336***	−1.337***
		(−4.78)	(−4.78)
Growth		−0.781***	−0.782***
		(−12.22)	(−12.26)
Cash		−0.164	−0.160
		(−0.63)	(−0.61)
OC		−0.003	−0.003
		(−0.79)	(−0.81)
Gender		−0.057	−0.061
		(−0.40)	(−0.43)
Age		−0.005	−0.005
		(−0.97)	(−1.05)
Subsidy		0.326***	0.326***
		(5.71)	(5.71)
_cons	−0.722***	−1.021	−1.026
	(−4.05)	(−0.61)	(−0.60)
Ind	Yes	Yes	Yes
Year	Yes	Yes	Yes
adj. *R*^2^	0.2479	0.2859	0.2868
*N*	20906	20906	20906

Note: *, ** and *** indicate statistical significance at the 10% level, 5% level and 1% level, respectively.

The results of the three regression models presented in Table 3 indicate that the regression coefficients for PID are 0.591, 0.707, and 0.870, respectively, all of which are statistically significant at the 5% or 1% level. This suggests a significant positive correlation between inside directors and corporate R&D investment. As the proportion of non-CEO inside director increases, the ratio of corporate R&D expenditure to sales revenue becomes greater. Therefore, the results of the regression analysis support the research hypothesis H1, indicating that inside directors contribute to the promotion of corporate R&D investment. The underlying reason is that inside directors can effectively enhance the corporate governance quality in the realm of technological innovation. Specifically, inside directors can effectively supervise the insufficient innovative behaviors of the CEO by reducing the degree of information asymmetry between the board and management, thereby enhancing the level of corporate R&D investment.

Furthermore, the results of the regression model presented in column (3) of Table 3 indicate that the coefficient for CEO equity incentives (EI) is 0.020, which is significantly positive at a level approaching 10% (with a T-value of 1.63, slightly below 1.65). This finding generally confirms the hypothesis that CEO equity incentives facilitate the alleviation of agency conflicts, thereby promoting corporate R&D investments [56]. Meanwhile, the coefficient of the interaction term between inside directors and equity incentives (PID×EI) is −0.078, which is statistically significant at the 10% level. This indicates that CEO equity incentives diminish the positive effect of inside directors on corporate R&D investment, thereby validating the research hypothesis H2 of this paper.

**Fig 1:**
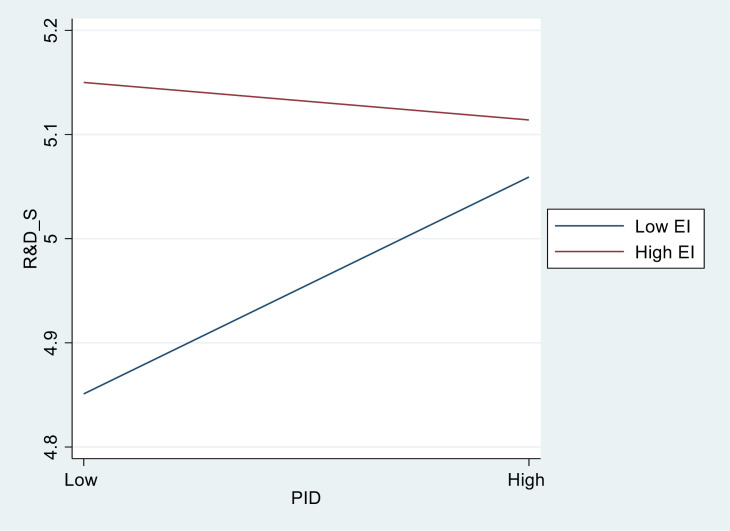
Moderating effect of CEO equity incentives.

To more clearly illustrate the relationship among CEO equity incentives (EI), inside directors (PID), and corporate R&D investment (R&D_S), we have constructed a moderation effect diagram. As illustrated in Fig 1, when the CEO receives a high level of equity incentives, the level of corporate R&D investment is relatively high, and inside directors do not have a significant promotive effect. Conversely, when the CEO receives a low level of equity incentives, the level of corporate R&D investment is comparatively low, and inside directors exhibit a significant promotive effect. CEO equity incentives help alleviate agency conflict within the firm, thereby increasing their willingness to undertake innovative risks and invest more in R&D projects. In this context, as the agency issues in the realm of technological innovation have been effectively addressed, the supervisory effect of inside directors will struggle to exert its original governance function, thereby no longer exerting a significant positive influence on corporate R&D investments.

In summary, the regression results simultaneously support the research hypotheses H1 and H2. In the face of the agency issue arising from insufficient innovation, inside directors can play a positive governance role, thereby enhancing the level of corporate R&D investments. At the same time, while CEO equity incentives positively influence corporate R&D investment, they diminish the promotive role of inside directors in this investment. Therefore, there exists a substitution relationship between CEO equity incentives and inside directors in terms of the effects of innovation governance.

### 4.3. Robustness tests

#### (1) Replacing the regression method.

Due to the non-negative values of the dependent variable (R&D_S) in this study, it is classified as a limited dependent variable, characterized by left truncation data (i.e., all values are greater than or equal to 0). The application of conventional regression methods may yield inconsistent and biased estimation results. The Tobit regression model, proposed by Tobin [57], is capable of effectively addressing regression issues involving limited dependent variable. Therefore, we draw upon the research conducted by Chang et al. [21] and employ the Tobit regression method as a substitute for the original model to test the robustness of the research findings. In the regression analysis, in addition to the proportion of non-CEO inside directors (PID), we also employed the number of non-CEO inside directors (NID) as a metric for the independent variable to enhance the robustness of the research findings. The regression results are presented in Table 4.

**Table 4:** Robustness test: Tobit regression method.

Variables	(1)	(2)	(3)	(4)
	R&D_S	R&D_S	R&D_S	R&D_S
PID	0.685***	0.850***		
	(3.87)	(4.49)		
NID			0.080***	0.096***
			(3.96)	(4.48)
EI		0.019**		0.016**
		(2.35)		(2.10)
PID×EI		−0.077***		
		(−2.65)		
NID×EI				−0.008**
				(−2.36)
Size	0.066**	0.066**	0.063*	0.064*
	(1.98)	(1.98)	(1.90)	(1.90)
Level	−1.289***	−1.291***	−1.289***	−1.292***
	(−9.04)	(−9.05)	(−9.04)	(−9.06)
Growth	−0.790***	−0.792***	−0.791***	−0.791***
	(−19.71)	(−19.75)	(−19.73)	(−19.75)
Cash	−0.204	−0.201	−0.207	−0.201
	(−1.25)	(−1.24)	(−1.27)	(−1.23)
OC	−0.003	−0.003	−0.003	−0.003
	(−1.19)	(−1.22)	(−1.17)	(−1.19)
Gender	−0.058	−0.061	−0.058	−0.061
	(−0.67)	(−0.71)	(−0.67)	(−0.70)
Age	−0.005	−0.005*	−0.005	−0.005*
	(−1.63)	(−1.72)	(−1.63)	(−1.71)
Subsidy	0.312***	0.313***	0.313***	0.313***
	(11.70)	(11.71)	(11.71)	(11.72)
_cons	−2.667***	−2.733***	3.135***	3.118***
	(−3.25)	(−3.32)	(8.52)	(8.47)
Ind	Yes	Yes	Yes	Yes
Year	Yes	Yes	Yes	Yes
Wald chi2	3593.05	3602.05	3593.56	3601.13
*N*	20906	20906	20906	20906

Note: *, ** and *** indicate statistical significance at the 10% level, 5% level and 1% level, respectively.

Column (1) and Column (2) in Table 4 present the regression results when the independent variable is PID. The regression coefficient of PID in column (1) is 0.685 and is statistically significant at the 1% level, indicating that a higher proportion of inside directors is associated with an increase in corporate R&D investment. In column (2), the regression coefficients for EI and PID×EI are 0.019 and −0.077, respectively, which are statistically significant at the 5% and 1% levels. This indicates that CEO equity incentives positively influence corporate R&D investment while simultaneously diminishing the promotive effect of the proportion of inside directors on such investment.

Column (3) and Column (4) in Table 4 present the regression results when the independent variable is NID. The regression coefficient of NID in column (3) is 0.080 and is statistically significant at the 1% level, indicating that a greater number of inside directors is associated with an increase in corporate R&D investment. In column (4), the regression coefficients for EI and NID×EI are 0.016 and −0.008, respectively, which are statistically significant at the 5% levels. This indicates that CEO equity incentives positively influence corporate R&D investment while simultaneously diminishing the promotive effect of the number of inside directors on such investment.

The aforementioned regression results further support the research hypotheses H1 and H2, indicating that inside directors can promote corporate R&D investment, while CEO equity incentives significantly diminish this promotive effect.

#### (2) Lagging independent variables.

In order to prevent possible reverse causality issues in the regression analysis, we draw upon the research methodology of Oruganti et al [58] by lagging both the independent and control variables by one year to examine the effect of inside directors from the previous year on the R&D investment in the current year. The re-regression results are shown in Table 5.

**Table 5:** Robustness analysis: Lagging independent variables.

Variables	(1)	(2)	(3)	(4)
	R&D_S	R&D_S	R&D_S	R&D_S
L.PID	0.382**	0.521**		
	(1.98)	(2.53)		
L.NID			0.044**	0.057**
			(1.99)	(2.45)
L.EI		0.021**		0.018**
		(2.42)		(2.21)
L.PID×EI		−0.070**		
		(−2.25)		
L.NID×EI				−0.007**
				(−1.99)
L.Size	0.267***	0.264***	0.265***	0.262***
	(7.24)	(7.11)	(7.20)	(7.07)
L.Level	−2.128***	−2.130***	−2.128***	−2.132***
	(−13.62)	(−13.63)	(−13.62)	(−13.64)
L.Growth	−0.501***	−0.502***	−0.501***	−0.502***
	(−11.42)	(−11.45)	(−11.43)	(−11.44)
L.Cash	0.324*	0.336*	0.323*	0.337*
	(1.85)	(1.91)	(1.84)	(1.92)
L.OC	−0.005**	−0.005**	−0.005**	−0.005**
	(−2.20)	(−2.21)	(−2.18)	(−2.21)
L.Gender	−0.083	−0.087	−0.083	−0.086
	(−0.86)	(−0.91)	(−0.86)	(−0.90)
L.Age	−0.002	−0.003	−0.002	−0.002
	(−0.54)	(−0.75)	(−0.53)	(−0.74)
L.Subsidy	0.253***	0.253***	0.253***	0.253***
	(8.98)	(8.99)	(8.98)	(8.99)
_cons	−4.336***	−4.265***	−4.305***	−4.235***
	(−4.78)	(−4.67)	(−4.75)	(−4.64)
Ind	Yes	Yes	Yes	Yes
Year	Yes	Yes	Yes	Yes
Wald chi2	2221.00	2229.62	2220.62	2228.26
*N*	17684	17684	17684	17684

Note: *, ** and *** indicate statistical significance at the 10% level, 5% level and 1% level, respectively.

Columns (1) and (2) present the regression results when the independent variable is PID, while Columns (3) and (4) display the regression results when the independent variable is NID. It can be observed that regardless of whether the independent variable is PID or NID, the coefficients for inside directors are significantly positive at the 5% level. Meanwhile, the coefficients for the interaction term between inside directors and equity incentives are significantly negative at the 5% level as well. This regression result further validates the positive influence of inside directors on corporate R&D investments, as well as the negative moderating effect of CEO equity incentives. Our research findings remain consistent.

#### (3) Increase control variables.

In addition, to mitigate the issue of omitted variable bias, we incorporated control variables related to corporate governance into the regression model, in addition to controlling for firm characteristics, CEO characteristics, and government funding. Specifically, the newly added control variables include CEO duality (Duality), management shareholding ratio (M_share), and institutional investor shareholding ratio (I_share).

The results of the regression analysis are presented in Table 6. Regardless of whether the independent variable is PID or NID, the regression coefficients of the independent variables are significantly positive at the 1% level, while the regression coefficients of the interaction terms (PID×EI and NID×EI) are significantly negative at the 1% and 5% levels, respectively. This indicates that our research findings remain valid even after the inclusion of control variables related to corporate governance.

**Table 6:** Robustness analysis: Increase control variables.

Variables	(1)	(2)	(3)	(4)
	R&D_S	R&D_S	R&D_S	R&D_S
PID	0.573***	0.741***		
	(3.12)	(3.80)		
NID			0.068***	0.084***
			(3.26)	(3.81)
EI		0.011		0.008
		(1.33)		(0.97)
PID×EI		−0.075***		
		(−2.59)		
NID×EI				−0.007**
				(−2.29)
Size	0.054	0.062*	0.052	0.061*
	(1.57)	(1.78)	(1.52)	(1.74)
Level	−1.235***	−1.231***	−1.235***	−1.233***
	(−8.61)	(−8.58)	(−8.61)	(−8.60)
Growth	−0.800***	−0.803***	−0.801***	−0.803***
	(−19.91)	(−19.98)	(−19.92)	(−19.97)
Cash	−0.281*	−0.300*	−0.283*	−0.299*
	(−1.71)	(−1.82)	(−1.72)	(−1.82)
OC	−0.006**	−0.006**	−0.005**	−0.006**
	(−2.29)	(−2.36)	(−2.25)	(−2.32)
Gender	−0.075	−0.077	−0.075	−0.076
	(−0.86)	(−0.89)	(−0.86)	(−0.88)
Age	−0.007**	−0.007**	−0.007**	−0.007**
	(−2.27)	(−2.17)	(−2.29)	(−2.18)
Subsidy	0.310***	0.311***	0.310***	0.311***
	(11.62)	(11.64)	(11.63)	(11.65)
Duality	0.041	0.044	0.045	0.050
	(0.79)	(0.83)	(0.86)	(0.94)
M_share	0.652***	0.759***	0.654***	0.759***
	(3.17)	(3.42)	(3.18)	(3.43)
I_share	0.004***	0.004***	0.004**	0.004**
	(2.60)	(2.63)	(2.55)	(2.55)
_cons	−1.062	−1.278	−1.023	−1.245
	(−1.26)	(−1.49)	(−1.21)	(−1.45)
Ind	Yes	Yes	Yes	Yes
Year	Yes	Yes	Yes	Yes
Wald chi2	3617.92	3627.69	3618.80	3626.96
*N*	20906	20906	20906	20906

Note: *, ** and *** indicate statistical significance at the 10% level, 5% level and 1% level, respectively.

#### (4) System GMM analysis for dynamic panel model.

Due to the continuous nature of technological innovation, which entails a certain degree of “inertia,” corporate R&D investments are influenced not only by various contemporaneous factors but also by the level of prior R&D investment. Therefore, we introduce a lagged term for R&D investment (L.R&D_S) based on the original regression model, establishing a dynamic panel model to address the issue of innovation continuity. At the same time, considering the endogeneity issues, we conduct regression analysis using the System Generalized Method of Moments (SYS-GMM), following the studies by Xu et al [59] and Tian et al [60]. This method effectively addresses issues related to omitted variables, measurement error, and other endogeneity concerns, resulting in superior regression outcomes.

As shown in Table 7, after incorporating the lagged term of corporate R&D investment (L.S&D_S), the results of the system GMM regression indicate that the coefficients of L.R&D_S are approximately 0.7 and statistically significant at the 1% level. This finding corroborates our expectation that corporate R&D investment exhibits a strong continuity. Meanwhile, we also found that after controlling for the lagged dependent variable, regardless of whether the independent variable is PID or NID, the coefficients of the independent variables are significantly positive at the 1% level, while the coefficients of the interaction terms (PID×EI and NID×EI) are significantly negative at the 5% level. This indicates that inside directors can promote corporate R&D investment, while CEO equity incentives significantly diminish this promotive effect. It can be seen that after employing the dynamic panel model with system GMM analysis, our research findings remain valid.

**Table 7:** Robustness analysis: System GMM analysis for dynamic panel model.

Variables	(1)	(2)	(3)	(4)
	R&D_S	R&D_S	R&D_S	R&D_S
L.R&D_S	0.696***	0.699***	0.701***	0.702***
	(28.23)	(28.91)	(28.71)	(29.27)
PID	6.962***	6.122***		
	(7.20)	(6.34)		
NID			0.760***	0.639***
			(6.52)	(5.60)
EI		0.056**		0.057**
		(2.09)		(2.46)
PID×EI		−0.264**		
		(−2.38)		
NID×EI				−0.030**
				(−2.57)
Size	0.156**	0.166***	0.129**	0.139**
	(2.46)	(2.60)	(2.07)	(2.21)
Level	−0.858***	−0.846***	−0.926***	−0.901***
	(−2.90)	(−2.93)	(−3.16)	(−3.15)
Growth	−1.070***	−1.063***	−1.067***	−1.056***
	(−13.94)	(−14.01)	(−13.92)	(−13.99)
Cash	−0.543*	−0.487	−0.599*	−0.497
	(−1.66)	(−1.52)	(−1.84)	(−1.56)
OC	0.013**	0.013***	0.014**	0.015***
	(2.40)	(2.69)	(2.56)	(3.04)
Gender	−0.049	−0.080	−0.085	−0.110
	(−0.25)	(−0.42)	(−0.45)	(−0.59)
Age	−0.010*	−0.008	−0.008	−0.007
	(−1.67)	(−1.43)	(−1.43)	(−1.33)
Subsidy	0.153***	0.153***	0.155***	0.154***
	(2.80)	(2.85)	(2.85)	(2.87)
_cons	−3.547**	−3.752***	−2.855**	−3.053**
	(−2.50)	(−2.60)	(−2.04)	(−2.15)
Ind	Yes	Yes	Yes	Yes
Year	Yes	Yes	Yes	Yes
AR(2)	0.146	0.112	0.142	0.109
Wald chi2	41091.83	46560.70	41542.42	47687.28
*N*	17684	17684	17684	17684

Note: *, ** and *** indicate statistical significance at the 10% level, 5% level and 1% level, respectively.

### 4.3. Endogeneity tests

Due to sample selection bias, the study of the relationship between inside directors and corporate R&D investment may encounter endogeneity issues. Technological innovation is characterized by confidentiality and concealment. In order to win in the market competition, many firms may strategically hide information related to technological innovation and R&D investment, which could lead to self-selection bias. Therefore, the non-random selection of samples may represent an endogenous issue that cannot be overlooked in this study.

The Heckman two-stage model was proposed by Heckman [61], and is primarily utilized to address the issue of sample selection bias [62]. To this end, we draw upon the research of Chang et al [21], and endeavor to address this concern of endogeneity by employing the Heckman two-stage regression model. Specifically, the detailed process of this model encompasses two stages. In the first stage, we use the Probit Selection Model to predict whether a firm will disclose R&D investment, and calculate the “Inverse Mills Ratio” (IMR) through regression analysis. In the second stage, we incorporate the “Inverse Mills Ratio” (IMR) as an additional control variable into Eq. (1) to rectify the endogeneity issue arising from sample selection bias. Meanwhile, in order to mitigate the endogeneity issues arising from reverse causality, we apply a one-period lagged treatment to both the independent variables and the control variables.

To present the disclosure situation of corporate R&D investment, we use R&D_M (including missing values) as the dependent variable, with the regression results shown in Table 8. First of all, the regression coefficients of the “Inverse Mills Ratio” (L.IMR) from columns (1) to (4) are all significantly positive at the 1% statistical level, indicating that there is indeed endogeneity problem of non-random sample selection in this study. Secondly, among all regression models, the regression coefficients of the independent variables (L.PID and L.NID) are also significantly positive at the 1% statistical level, indicating that inside directors can significantly promote corporate R&D investment. Finally, the interaction terms between inside directors and equity incentives (L.PID×EI and L.NID×EI) exhibit significantly negative coefficients at the 5% and 1% levels, respectively, indicating that CEO equity incentives diminish the promotive effect of inside directors on R&D investment. Taken together, after addressing the issue of sample selection bias, the Heckman two-stage regression model further validates our findings and alleviates the concerns that endogeneity issue will driving the main results.

**Table 8:** Endogeneity tests: Heckman two-stage regression.

Variables	(1)	(2)	(3)	(4)
	R&D_M	R&D_M	R&D_M	R&D_M
L.PID	1.933***	1.666***		
	(5.02)	(4.50)		
L.NID			0.175***	0.161***
			(4.37)	(4.16)
L.EI		0.067***		0.073***
		(4.50)		(5.73)
L.PID×EI		−0.121**		
		(−2.12)		
L.NID×EI				−0.018***
				(−3.01)
L.Size	0.125**	0.089*	0.098*	0.068
	(2.26)	(1.81)	(1.95)	(1.51)
L.Level	−4.600***	−4.351***	−4.509***	−4.268***
	(−12.19)	(−13.00)	(−12.90)	(−13.73)
L.Growth	−0.177	−0.211**	−0.175*	−0.211**
	(−1.55)	(−2.04)	(−1.65)	(−2.20)
L.Cash	2.459***	2.502***	2.451***	2.504***
	(6.99)	(7.90)	(7.53)	(8.49)
L.OC	−0.012***	−0.012***	−0.012***	−0.012***
	(−3.96)	(−4.56)	(−4.26)	(−4.92)
L.Gender	0.143	0.126	0.129	0.114
	(0.87)	(0.85)	(0.85)	(0.83)
L.Age	0.005	0.003	0.006	0.003
	(0.90)	(0.51)	(1.07)	(0.64)
L.Subsidy	1.160***	1.147***	1.159***	1.144***
	(20.80)	(22.84)	(22.45)	(24.49)
_cons	−3.857***	−2.813**	−3.092**	−2.224*
	(−2.61)	(−2.15)	(−2.30)	(−1.86)
L.*IMR*	5.189***	4.675***	4.807***	4.351***
	(5.55)	(5.62)	(5.56)	(5.64)
Ind	Yes	Yes	Yes	Yes
Year	Yes	Yes	Yes	Yes
Wald chi2	2502.69	3081.48	2915.41	3558.89
*N*	16943	16943	16943	16943

Note: *, ** and *** indicate statistical significance at the 10% level, 5% level and 1% level, respectively.

## 5. Additional analysis: Heterogeneity tests

The innovative governance effects of inside directors are influenced by the corporate environment, the moral hazard of the CEO, and the governance efficiency of the board of directors. Existing research indicates that there are significant differences in attitudes and capabilities for technological innovation based on the nature of the firm, the gender and age of the CEO, and the size of the board [63,64]. Therefore, it is essential to further analyze the heterogeneity of the impact of inside directors on corporate R&D investment in order to clarify the boundary conditions under which inside directors exert their innovative governance effects.

Specifically, we categorize all research samples into state-owned enterprises (SOEs) and non-state-owned enterprises (non-SOEs) based on whether the actual controller belongs to the Chinese government (i.e., the nature of firms). We further classify all research samples into male CEOs (male) and female CEOs (female) according to the gender of the CEO. Additionally, we differentiate between older CEOs (older) and younger CEOs (younger) based on whether the CEO’s age exceeds the mean (49.95). Lastly, we categorize the research samples into large boards (large) and small boards (small) based on whether the board size exceeds the mean (8.40). On this basis, we examine the promotive effect of inside directors on corporate R&D investment to analyze the variability of governance effects exhibited by inside directors across different subsamples.

In the process of regression analysis, we employed the percentage of R&D expenditure relative to sales (R&D_S) as the dependent variable, while the proportion of inside directors (PID) was utilized as the independent variable. Consistent with the aforementioned approach, in order to mitigate the issue of endogeneity, we not only lag the independent and control variables but also employed the Heckman two-stage regression model. The regression results are presented in Table 9.

**Table 9:** Additional analysis: Heterogeneity tests.

Variables	The nature of firms		The gender of CEOs		The age of CEOs		Board size	
	(1) SOEs	(2)non-SOEs	(3) male	(4) female	(5)older	(6)younger	(7) large	(8) small
L.PID	−0.935**	2.122***	2.475***	−1.465	3.097**	0.443	0.117	3.390***
	(−2.11)	(5.44)	(4.16)	(−0.85)	(2.11)	(1.19)	(0.15)	(3.85)
L.Size	−0.072	0.205***	0.206**	−0.335*	0.287	−0.095*	−0.180	0.041
	(−1.06)	(3.51)	(2.29)	(−1.83)	(1.13)	(−1.70)	(−1.07)	(0.53)
L.Level	−2.721***	−4.825***	−5.105***	−2.096	−5.882***	−2.995***	−2.480***	−5.347***
	(−6.49)	(−12.04)	(−8.38)	(−1.56)	(−3.07)	(−8.84)	(−2.60)	(−5.21)
L.Growth	0.011	−0.227**	−0.093	−0.381	0.332	−0.343***	−0.154	−0.216
	(0.08)	(−2.04)	(−0.58)	(−1.19)	(0.78)	(−2.89)	(−1.49)	(−1.03)
L.Cash	0.340	3.186***	2.530***	1.356	2.859***	2.709***	2.043***	3.126***
	(0.63)	(9.28)	(5.13)	(1.43)	(2.66)	(7.53)	(6.62)	(4.88)
L.OC	0.001	−0.013***	−0.012***	−0.002	−0.005	−0.016***	−0.012***	−0.006
	(0.20)	(−4.21)	(−2.80)	(−0.10)	(−0.42)	(−5.00)	(−3.52)	(−0.61)
L.Gender	0.557**	0.066	0.000	0.000	−0.302	0.024	−0.081	−0.432
	(2.09)	(0.44)	(.)	(.)	(−0.60)	(0.14)	(−0.34)	(−1.45)
L.Age	−0.022**	0.013**	−0.002	0.068***	−0.084	0.135***	0.006	0.013
	(−2.51)	(2.37)	(−0.21)	(3.47)	(−1.53)	(4.93)	(1.11)	(1.16)
L.Subsidy	0.748***	1.303***	1.178***	1.153***	1.401***	1.073***	1.051***	1.350***
	(12.19)	(22.84)	(15.08)	(6.13)	(7.18)	(18.64)	(21.60)	(12.70)
_cons	1.300	−5.442***	−6.021**	5.911	−6.270	−1.485	5.821	−4.392*
	(0.63)	(−3.52)	(−2.44)	(1.18)	(−1.04)	(−1.38)	(1.19)	(−1.90)
L.*IMR*	2.835**	4.323***	6.971***	−1.588	10.716*	−2.207***	−1.827	5.796**
	(2.09)	(4.57)	(4.01)	(−0.61)	(1.90)	(−3.56)	(−0.58)	(2.47)
Ind	Yes	Yes	Yes	Yes	Yes	Yes	Yes	Yes
Year	Yes	Yes	Yes	Yes	Yes	Yes	Yes	Yes
Wald chi2	1683.51	2859.41	1331.11	346.40	319.39	3037.78	3574.04	894.69
*N*	5480	15426	19546	1360	11425	9481	12562	8344

Note: *, ** and *** indicate statistical significance at the 10% level, 5% level and 1% level, respectively.

### 5.1. The nature of firms

The regression results from columns (1) and (2) of Table 9 indicate that, for SOEs, the regression coefficient for inside directors (L.PID) is −0.935, which is statistically significant at the 5% level. For non-SOEs, the regression coefficient of inside directors (L.PID) is 2.122, which is statistically significant at the 1% level. It is evident that inside directors in SOEs may inhibit corporate R&D investment, whereas inside directors in non-SOEs are able to significantly improve the level of corporate R&D investment. In other words, the promotive effect of inside directors on corporate R&D investment is applicable only to non-SOEs, and not to SOEs.

The reason for this result may be that compared with non-SOEs, CEOs of SOEs do not exhibit a lack of innovation, rather, they may be inclined towards excessive innovation. On the one hand, SOEs have dual economic and political missions. In the context of the Chinese government advocating an innovation-driven development strategy, CEOs of SOEs will tend to increase R&D investment to actively respond to government needs [65]. On the other hand, SOEs have significant advantages in obtaining innovative resources. They often have priority in obtaining bank loans and enjoy more government support [66]. Therefore, SOEs have more innovation resources and less innovation risks in R&D investment. In this case, compared with non-SOEs, CEOs of SOEs may have a stronger willingness for technological innovation. Correspondingly, the supervisory effect of inside directors not only fails to promote corporate R&D investment but may, in fact, inhibit it.

### 5.2. The gender of CEOs

The regression results from columns (3) and (4) of Table 9 indicate that, for the sample of male CEOs, the regression coefficient for inside directors (L.PID) is 2.475, which is statistically significant at the 1% level. In contrast, for the sample of female CEOs, the regression coefficient for inside directors (L.PID) is −1.465, and it does not reach a statistically significant level. It is evident that when the CEO is male, inside directors can significantly improve the level of corporate R&D investment; however, when the CEO is female, the impact of inside directors is not significant. Therefore, the promotive effect of inside directors on corporate R&D investment is applicable only to male CEOs, and not to female CEOs.

The underlying reason is that, compared to male CEOs, female CEOs may experience a smaller agency conflict with the board of directors, thereby limiting the opportunity for inside directors to exert their supervisory effects. On the one hand, according to the ethical sensitivity theory [67], female leaders are more inclined to consider the interests of other stakeholders, exhibiting a more stringent ethical value system compared to their male counterparts, and are less likely to engage in self-serving behaviors [68]. On the other hand, compared to male CEOs, female CEOs exert a more positive influence on corporate information disclosure, resulting in high-quality reports that are more comprehensive, authentic, and readable [69]. This helps to mitigate the information asymmetry between the CEO and the board, thereby reducing the information transmission role of inside directors. Therefore, for female CEOs, not only will the issue of information asymmetry be alleviated, but the occurrence of opportunistic behaviors will also be diminished. In this context, inside directors are unable to exert their supervisory effect, resulting in a failure to significantly improve the level of corporate R&D investment.

### 5.3. The age of CEOs

The regression results from columns (5) and (6) of Table 9 indicate that the coefficient for inside directors (L.PID) in the sample of older CEOs is 3.097, which is statistically significant at the 5% level. In contrast, the coefficient for inside directors (L.PID) in the sample of younger CEOs is 0.443, which does not reach statistical significance. It is evident that for older CEOs, inside directors can significantly improve the level of corporate R&D investment, whereas for younger CEOs, the role of inside directors is not significant. Therefore, the promotive effect of inside directors on corporate R&D investment is applicable only to older CEOs, and not to younger CEOs.

The underlying reason for this phenomenon may be related to the CEO’s career horizon [70]. The career horizon refers to the remaining years of tenure for a CEO prior to retirement. Older CEOs typically possess a shorter career horizon, whereas younger CEOs tend to have a longer career horizon. The project cycle for R&D investments is relatively long and characterized by significant uncertainty, which may result in an inability to generate corresponding returns in the short term [71]. Compared to younger CEOs, older or near-retirement CEOs are more likely to engage in opportunistic behaviors characterized by insufficient investment in R&D, in order to enhance corporate performance during their tenure. Research has indicated that older CEOs exhibit significantly lower expenditures on corporate R&D projects compared to their counterparts in other firms [72]. In contrast, for younger CEOs, there is no significant issue of inadequate innovation, as they possess a longer career horizon and do not shy away from engaging in long-term technological innovation activities. In this context, inside directors are not required to engage in supervisory activities, and thus cannot significantly promote corporate R&D investments.

### 5.4. Board size

The regression results from columns (7) and (8) of Table 9 indicate that for large boards, the regression coefficient for inside directors (L.PID) is 0.117, which does not reach a statistically significance. In contrast, for small boards, the regression coefficient for inside directors (L.PID) is 3.390, and it is statistically significant at the 1% level. It can be observed that the influence of inside directors in large boards on corporate R&D investment is not significant, whereas inside directors in small boards can significantly improve the level of corporate R&D investment. Therefore, the promotive effect of inside directors on corporate R&D investment is applicable only to small boards, and not to large boards.

The governance efficiency of the board is closely related to its size, small boards are often perceived as more effective compared to large boards. Specifically, large boards often encounter issues of poor communication and slow progress in the decision-making process, making it challenging to effectively monitor the opportunistic behavior of the CEO [73]. Research indicates that large boards tend to have a negative impact on corporate R&D investments [64]. In contrast, small boards facilitate high-quality discussions among all board members and enhance their level of engagement in the decision-making process. In general, the optimal size for a board to effectively fulfill its governance role should not exceed nine members [74]. From the above analysis, it can be concluded that large boards of directors, due to their inefficiency in governance, are unable to effectively supervise the insufficient innovation of the CEO. Only in small boards with high governance efficiency can inside directors exert their governance effects and promote corporate R&D investment.

## 6. Discussion

Regarding the relationship between the board of directors and technological innovation, previous research has predominantly explored the impact of independent directors and outside directors on corporate R&D investment from the perspective of board independence [23], while neglecting the potential role of inside directors. Our paper addresses this research gap by thoroughly investigating the impact of inside directors on corporate R&D investment, resulting in a series of research findings.

Firstly, the research findings indicate that inside directors can promote corporate R&D investments. This finding reshapes the significant role of inside directors in corporate technological innovation, reversing the previously held negative perception of inside directors as biased, unreliable, and contrary to shareholder interests [75]. Previous studies suggest that to mitigate the opportunistic behavior of CEOs, it is generally necessary to enhance the independence of the board in order to strengthen the supervision on the CEO, thereby promoting corporate R&D investment [6]. Our research findings indicate that inside directors, as one of the components of the board of directors, also play a significant role in governance. Especially in the realm of technological innovation, inside directors possess the prerequisite, motivation, and capability to supervise the insufficient innovative behaviors of the CEO, given their information advantages. This, in turn, contributes to the promotion of corporate R&D investments.

Our findings indirectly supports the perspective of the “independence paradox” [13]. Without inside directors providing the board with internal information, the board may become susceptible to manipulation by management, thereby impeding its ability to fulfill effective governance functions. Meanwhile, this research finding provides new evidence on the positive governance role of inside directors. Recent studies show that inside directors can reduce the information asymmetry between the board and management, so it plays an important role in CEO turnovers [15], CSR management [16], and internal control efficiency [17]. In line with the above studies, our study supports the positive governance role of inside directors by demonstrating their promotive effect on corporate R&D investment.

Secondly, this study also found that CEO equity incentives may undermine the positive impact of inside directors on corporate R&D investments. To prevent opportunistic behavior arising from insufficient innovation by management, agency theory posits that two governance mechanisms can be employed: board supervision and management incentives [24]. Our research indicates that CEO equity incentives contribute to promoting corporate R&D investment, however, they simultaneously diminish the influence of inside directors on R&D investment. In other words, both inside directors and CEO equity incentives can positively influence R&D investment, however, there exists a substitutive relationship between the two. The underlying reason is that when CEOs possess equity incentives, they are more likely to actively engage in R&D investments, thereby alleviating the agency conflicts in the realm of technological innovation, which consequently reduces the innovative governance effect of inside directors.

Previous research indicates that in the realm of internal control and earnings management, board supervision and CEO incentives can serve as complementary governance mechanisms [47,48]. This paper, based on the realm of technological innovation, reveals that inside board and CEO equity incentives serve as substitutive governance mechanisms for R&D investment, thereby enriching the existing research on governance bundling. Meanwhile, this finding also corroborates the conclusions of Lim [20] and Zona [19], who found that the positive impact of CEO restricted stock and stock option incentives on R&D investment may be attenuated by the vigilance and supervision from the board of directors. It is evident that the presence of multiple governance mechanisms in R&D investment does not yield a cumulative effect. Regulators should strike an appropriate balance between board supervision and CEO incentives to avoid excessive governance [26].

Thirdly, we also observed that the influence of inside directors on corporate R&D investment exhibits significant variation across different research samples. Specifically, the promotive effect of inside directors on R&D investment is significant only in the samples of non-SOEs, male CEOs, older CEOs, and small boards, while it is not significant in the samples of SOEs, female CEOs, younger CEOs, and large boards. The underlying reason is that, for SOEs, female CEOs, and young CEOs, the agency conflicts regarding innovation are not significant, leaving little space and opportunity for inside directors to exert governance roles. For large boards, the low governance efficiency undermines the governance capacity of inside directors.

Previous studies have indicated that SOEs, due to their political missions [65], female CEOs, who possess stricter ethical values [67], and younger CEOs, who have a longer career horizon [70], are all more likely to actively engage in technological innovation activities. Meanwhile, large boards are likely to exhibit inefficient governance due to poor communication and slow decision-making [64]. Our research findings not only enrich the existing research framework but also corroborate the results of the aforementioned literature.

## 7. Conclusion

Corporate governance advocated by traditional agency theory requires the continuous strengthening of the independence of the board, thereby maintaining vigilance against inside directors. Under the ongoing impetus of this notion, inside directors have become an endangered species, confined to a limited number of positions on corporate boards [7]. Based on panel data of 3,002 Chinese manufacturing listed firms from 2011 to 2021, we empirically test the positive impact of inside directors on corporate R&D investment, thereby providing new insights for optimizing board structure.

Our study finds that inside directors help constrain CEOs’ opportunistic behavior of insufficient innovation and significantly promote corporate R&D investment. It shows that inside directors are not the CEO’s “allies” but an important “governance force”. This study also finds that although CEO equity incentives are beneficial to promoting corporate R&D investment, it negatively moderates the positive impact of inside directors on R&D investment. The results indicate that the supervisory effect of inside directors and the incentive effect of the CEO both contribute to promoting corporate R&D investment, and that these effects are substitutive rather than complementary. Additional analysis indicates that the promotive effect of inside directors on corporate R&D investment is significant only within the samples of non-SOEs, male CEOs, older CEOs, and small boards. In contrast, this effect is not significant within the samples of SOEs, female CEOs, younger CEOs, and large boards. It indicates that the governance effect of inside directors does not apply to all firms, when the CEO does not exhibit moral hazard or when the board demonstrates lower supervisory efficiency, inside directors are unable to exert a positive influence on corporate R&D investment.

How to prevent opportunistic behavior related to insufficient innovation by CEOs is an important topic in corporate governance research. Previous research has predominantly focused on the influence of outside or independent directors on technological innovation, while neglecting the significant role of inside directors. Given the informational advantages and the influence of inside directors, this study delves into the impact of inside directors on corporate R&D investment, thereby contributing to the theoretical discourse in this area. Firstly, it contributes a novel perspective to the relevant research on the driving factors of corporate R&D investment. To the best of our knowledge, there is a paucity of research examining the relationship between inside directors and R&D investment. Our study contributes to filling the gaps in the relevant literature within this field. Secondly, our research simultaneously considers the supervisory effects of inside directors and the incentive effects of CEOs, and confirms their substitutive relationship in promoting R&D investment. This further enriches the research findings on governance bundling in technological innovation. Thirdly, through heterogeneity analysis, our study further clarifies the boundary conditions of the innovative governance effects of inside directors, thereby expanding the existing research framework.

Corporate R&D investment is the premise and foundation for technological innovation and an important guarantee for the country to achieve high-quality development. Under the current mainstream view that emphasizes board independence, our findings provide new evidence for reversing the negative image of inside directors, and have important practical significance for future corporate governance reforms.

First of all, from the perspective of board structure, we should fully understand the important role of inside directors in corporate governance. Managers who also serve as directors can alleviate information asymmetry and possess both the motivation and capability to supervise CEO’s opportunistic behavior and promote corporate R&D investment. Therefore, while maintaining the independence of the board, people must also pay attention to the important role of inside directors. Regulators should encourage or require firms to appropriately increase the number and proportion of non-CEO inside directors to enhance the innovative governance capabilities.

Secondly, from the perspective of governance portfolio, supervision mechanisms and incentive mechanisms should be comprehensively considered and appropriately configured. The research results show that CEO equity incentive and inside directors both contribute to promoting corporate R&D investment, however, there exists a substitutive relationship between the two. For firms with more CEO equity incentives, the governance role of inside directors is significantly reduced. Therefore, in terms of corporate R&D investment, multiple governance mechanisms do not yield an additive effect, regulators should strike an appropriate balance between inside board and CEO incentives to avoid the issue of excessive governance.

Finally, from the perspective of heterogeneity, the arrangement of inside directors should exhibit a certain degree of flexibility for different firms. Our research findings indicate that the influence of inside directors on corporate R&D investment exhibits significant variation across different samples. Therefore, regulators should refrain from adopting a one-size-fits-all approach when determining the board structure, and the configuration of inside directors should be tailored to the characteristics of the firm, the CEO, and the board of directors.

Due to the constraints imposed by our focus and data sources, this paper also has certain limitations. Firstly, due to the limitations arising from data availability, we were unable to empirically examine the intrinsic mechanisms through which inside directors influence R&D investment. Future research could explore the specific processes through which inside directors supervise the CEO, utilizing data such as the minutes of board meetings. Secondly, the research sample of this study is derived from China, where the government has imposed a limit on the number of inside directors (not to exceed 1/2 of the board size). This regulation may impact the generalizability of the findings of this paper. Future research could explore the governance effects of inside directors in other countries and regions, particularly in contexts where there are varying regulations regarding the number of inside directors.

## Supporting information

S1 Raw data All raw data used in this paper. (XLSX)
